# Revisiting the issue of access to medicines in Africa: Challenges and recommendations

**DOI:** 10.1002/puh2.9

**Published:** 2022-06-15

**Authors:** Yusuff Adebayo Adebisi, Ifechukwu Benedict Nwogu, Aishat Jumoke Alaran, Abubakar Olaitan Badmos, Adeboye Olakunle Bamgboye, Basirat Oluwadamilola Rufai, Osaretin Christabel Okonji, Malik O. Malik, John Oluwafemi Teibo, Samah Faisal Abdalla, Don Eliseo Lucero‐Prisno, Mohamed Samai, Wuraola Akande‐Sholabi

**Affiliations:** ^1^ Global Health Focus Africa Abuja Nigeria; ^2^ Department of Clinical Pharmacy and Pharmacy Administration Faculty of Pharmacy University of Ibadan Ibadan Nigeria; ^3^ The Comparative Health Outcomes Policy and Economics (CHOICE) Institute School of Pharmacy University of Washington Seattle Washington USA; ^4^ National Primary Healthcare Development Agency Abuja Nigeria; ^5^ Faculty of Pharmacy Obafemi Awolowo University Ile‐Ife Nigeria; ^6^ School of Pharmacy University of the Western Cape Cape Town South Africa; ^7^ Department of Biochemistry and Immunology Ribeirão Preto Medical School University of São Paulo Ribeirão Preto SP Brazil; ^8^ Faculty of Medicine University of Khartoum Sudan; ^9^ Department of Global Health and Development London School of Hygiene and Tropical Medicine and Health United Kingdom; ^10^ Department of Pharmacology and Therapeutics College of Medicine and Allied Health Sciences University of Sierra Leone Freetown Sierra Leone

**Keywords:** access to medicines, Africa, public health, Universal Health Coverage

## Abstract

**Background:**

Access to safe, effective, affordable, and quality medicines is an essential component of the right to health and is also one of the targets in the global development agenda. In this review article, we extensively discuss the challenges and issues surrounding access to medicines in the African region as well as provides recommendations for ensuring medicines security on the continent.

**Methods:**

We conducted narrative review with the use of data reported in published literature, reports, and grey literature available in African countries on topics pertaining access to medicines. The authors also snowballed further data to gather information for this review and narrative synthesis was conducted.

**Results:**

Africa faces a double burden of infectious and non‐communicable diseases and the need for effective universal access to medicines cannot be deemphasized. However, access to medicines on the continent is not without issues and challenges. Some of which are the high burden of infectious diseases and non‐infectious diseases, limited pharmaceutical industries and high costs of raw materials, overdependence on countries abroad for medicines, poor supply chain systems, lack of government investment in the pharmaceutical sector, unfavourable manufacturing conditions, limited health workforce, lack of sustainable health financing mechanisms, lack of infrastructures and technical know‐how, low investment on research and development, and circulation of fake and counterfeit medicines among others.

**Conclusion:**

This review reifies that access to medicines in Africa faces numerous challenges and it emphasizes the urgent need to address these issues as the continent geared towards strengthening its health systems for universal health coverage.

## Graphical abstract

Access to medicine remains a major challenge in Africa with peculiar implications for the achievement of Universal Health Coverage on the continent. We urge global and local stakeholders to strengthen access to medicine in Africa because, without access to quality, affordable and safe medicines, achieving equitable health for all is a mirage.

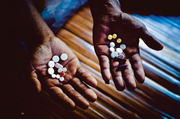

## BACKGROUND

The need to have access to safe and effective medicines is so important that it has been designated as a basic human right by the World Health Organization [1]. The relevance has been given further weight by its inclusion in the Sustainable Development Goals (SDGs) target 3.b [2]. Efforts to ensure access to medicines are mainly driven by an ethical imperative: people should not be denied access to life‐saving or health‐promoting interventions for unfair reasons, including economic or social causes [3]. Within the African context, certain limitations exist concerning their access to basic medicines [4, 5]. Many people living in Africa have a problem with accessing medicines and this undoubtedly contributes to the poor health metrics of most countries in the region [6]. Healthcare expenditures in many parts of Africa fall below what is required to achieve Universal Health Coverage (UHC) and many people have to resort to out‐of‐pocket payments for medicines [7]. The African region also has the highest burden of communicable diseases (like tuberculosis, HIV/AIDS, malaria) [8] and a significantly high burden of non‐communicable diseases such as diabetes and heart diseases [9]. These facts, therefore, suggest that access to medicines is a vital issue to discuss concerning UHC attainment in a continent with weak health systems [10, 11].

Access to medicines has been defined by the United Nations as ‘percentage of the population who have access to a minimum list of 20 essential medicines, which are continuously available and affordable at a health facility or medicines outlet, within 1 h walk from the patient's home’ [12]. This definition takes into consideration the basic components of access to medicines, that is, accessibility, affordability, availability, and acceptability [13]. Access to medicines takes into account the effectiveness of the supply chain with respect to supply location and location of the end‐user [13]. Considering that many parts of Africa are still classified as rural or semi‐urban areas, the efficiency of medicines supply could be influenced by geographical barriers, cultural barriers, and shortage of qualified medical personnel [14].

The African sub‐region relies heavily on the importation of medicines and raw materials for the manufacture of their medications [15, 16]. This could spell serious trouble for rural areas since they are typically the last to get access to any imported medicines or manufactured medicines due to their inaccessibility. This also means that changes to supply chain logistics or policies affecting the importation of the drugs or raw materials could cause scarcity and make medicines inaccessible. The acceptability of medicines correlates with patients’ familiarity with medicines [14] and this has implications for a region that is constantly receiving medicines from multiple sources internationally. Patients may refuse certain forms of treatment because they are unfamiliar, or due to religious and cultural reasons [17]. Similarly, drug distributors and importers (including community pharmacies) may refuse to import or stock certain drugs if they believe that their target population would not accept them. This could place a limit on the number of drugs that can be accessed by people in such an area. Availability of drugs borders on supply chain logistics with respect to demand and supply [13].

Despite acknowledging the need for affordable and accessible treatments, there is little evidence to demonstrate that African governments are intensely working on ensuring access of medicines to citizens. The affordability of medicines is another vital part of access to medicines and it correlates well with the prices of drugs and the income of end‐users. This paper elucidates the challenges further, and issues surrounding access to medicines in the African region as well as provides recommendations for ensuring medicine security on the continent.

## METHODS

**FIGURE 1:**
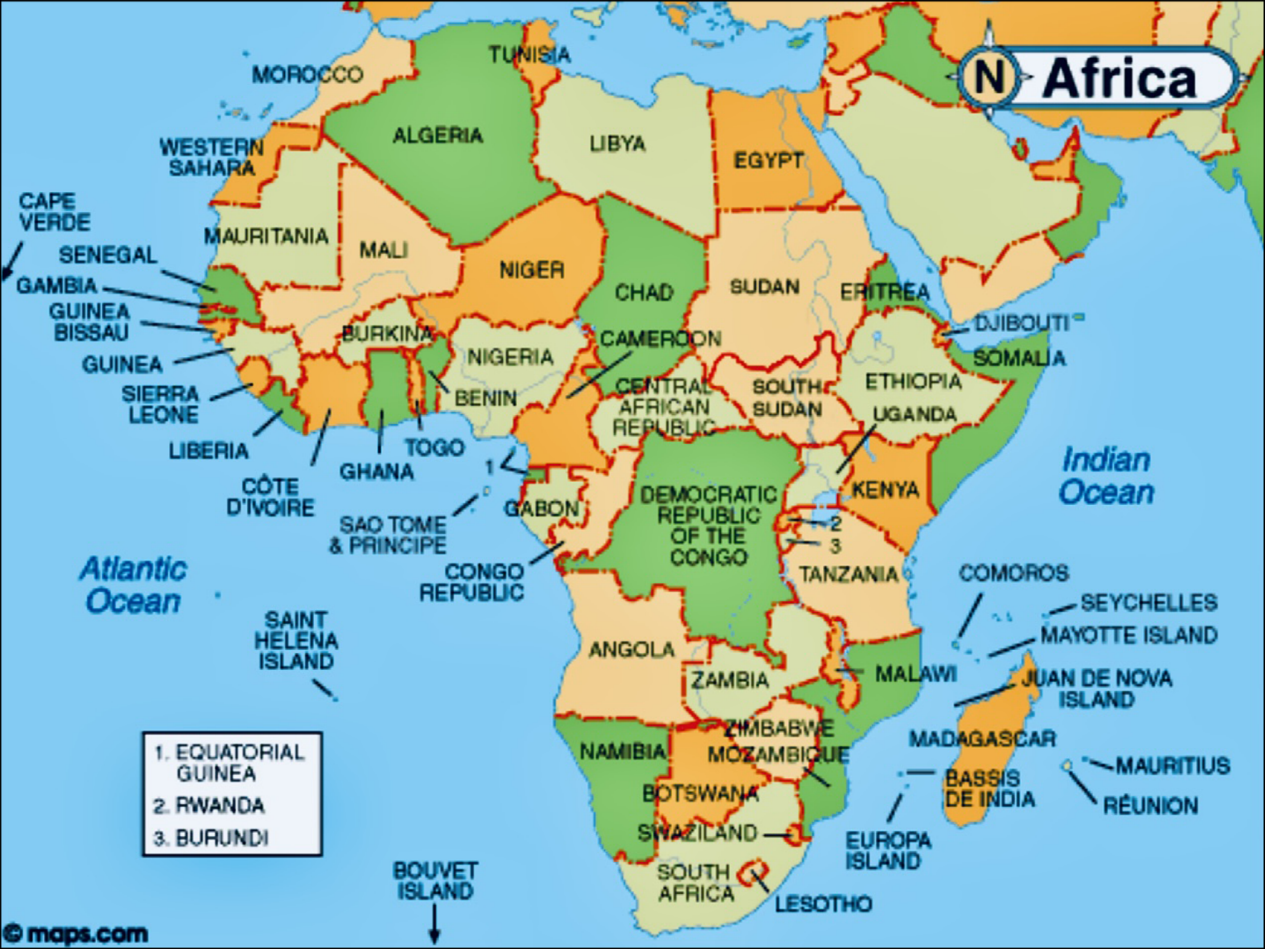
A map showing the countries in Africa (maps.com)

The methodological approach utilized in this narrative review is similar to what was used in some previous studies [18, 19]. We conducted narrative review with the inclusion of relevant papers published up to March 2022 and in English Language. Data reported in this study were gotten from published literature in African countries on topics pertaining access to medicines. The authors also snowballed further data to gather information for this review by reviewing the references of the included papers and narrative synthesis was conducted with theme of discussion identified across studies. Our data sources included journal articles from different e‐bibliographic databases, including MEDLINE, PubMed Central, PubMed, and Google Scholar. The search terms used include ‘Access to medicines’, ‘Medicine Access’, ‘Africa’, ‘Issues’, ‘Challenges’ ‘Efforts’. We also included the name of African countries in the search box to gather useful data and information (See Figure 1 for the map of Africa). The definition of access to medicines is seen across the literature in terms of medicines affordability, accessibility and availability.

The inclusion criteria include data sources that provide information regarding access to medicines in African countries and were published in English and up to March 2022 while the exclusion criterion was any other data sources that do not provide information regarding access to medicines in Africa. Four researchers (Y.A.A., I.B.N., A.J.A., A.O.B.) were involved in the independent review of literature to gather data for this study. The collected articles were managed using Endnote Reference Manager Software version X8 with a prior review of the title and abstract. In case of any duplication of references or disagreement, a consensus was reached through discussion. Additionally, to ensure the quality of data and correctness of facts (check and balance), the authors had a brainstorming session on this.

Supplementary data were also gathered from country reports, newsletters, commentaries, policy briefs and other reports as well as direct google search as it was inferred that some of the relevant sources are not published in peer‐reviewed academic journals because they are not empirical studies but policy papers. The extracted data were discussed narratively, with themes identified, to explore the aim of the study.

### **Challenges f**acing access to medic**ine in Africa**

We extensively discuss the challenges and issues associated to access to medicines in Africa.

### High burden of infectious diseases and non‐infectious diseases

Despite the feeble healthcare systems, African countries are challenged with a double burden of infectious diseases and non‐infectious diseases [20].  While communicable diseases still account for about two‐thirds of deaths in Africa, age‐specific death rates among men and women from chronic non‐communicable diseases are greater in sub‐Saharan Africa than in any region globally [21]. This has resulted in heavy demand for medicines for diseases treatment and prevention, making access to medicines on the continent considerably difficult for some patients, especially among the marginalized and vulnerable groups. This situation is worrisome and concerning in Africa because of the presence of limited pharmaceutical manufacturing capacity to cater to the continent medicine's needs [15]. In other words, the demand for medicines surpasses local drug manufacturing capacity in Africa [22] and the pervasive double burden of diseases is one of the possible drivers.

More so, the emergence of the COVID‐19 pandemic has resulted in a shortage of medicines across the continent due to the global travel bans and lockdowns, which affect medicines manufacturing and importation [23, 24, 25]. The urgent need to have access to medicines among the populace, due to the high diseases burden, is a potential route that can facilitate the circulation of fake and counterfeit medicines on the continent and the presence of porous supply chain systems worsen this further. This implies that, as the efforts to curb diseases incidence and prevalence intensify, there is a need to invest in solutions and efforts aimed at ensuring uninterrupted access to effective, safe, quality, and affordable medicines. To achieve this, localization of drug manufacturing in Africa is much‐needed.

### Limited pharmaceutical industries and high costs of raw materials

The dearth of drug manufacturing industries in Africa vis‐à‐vis the exorbitant cost of raw materials constitutes a huge limitation to the ease of access to drugs. It is disheartening that Africa accounts for only 3% of global production of drugs [26] despite bearing 24% of the global burden of disease and accounting for 11% of the world's population [27]. A 2019 McKinsey report noted that, for the 1.1 billion people in Africa, there are only roughly 375 drug‐producing industries, mostly in the northern region [28]. Those in sub‐Saharan Africa are clustered in nine out of 46 countries [28]. This is massively pale in comparison with China and India where 10,000 and 5000 drug manufacturers, respectively, serve 1.1 billion people [28].

In 2005, a World Bank research identified that Nigeria, South Africa, Kenya, and Zimbabwe have the industrial capacity that can be channelled to the production of medicines for domestic consumption or export [29]. However, they warned that if the economic cost of creating local production capacity is high or the quality of products is subliminal, this potential is prone to depleting [29]. The cost of production and the quality of drugs manufactured are hugely dependent on the availability of raw materials, including active pharmaceutical ingredients (the primary components in the manufacture of drugs). In Africa, raw materials and equipment needed for drug production are mostly imported, contributing significantly to the cost of production [30]. Thus, industries are forced to rely heavily on imports and thereby incur excessive expenditures. This directly translates to a severe reduction in both the quantity and quality of drugs produced. Additionally, this usually results in very small profit margins, discouraging investment and hampering the growth of industries.

The growth of local pharmaceutical industries also relies heavily on the availability of infrastructures such as transportation systems, communication networks, and constant power supply [31]. These are starkly unavailable in most resource‐limited settings of countries in Africa. Combined with its fragile economy and ridiculous level of sustained poverty, indigenous drug production faces tedious constraints [30]. The consequences thereof are suffered by the populace who have extremely limited access to essential and non‐essential drugs. There is no better time than now to promote more local production of medicines by private and government‐owned pharma companies as well as industries that manufacture active pharmaceutical ingredients.

### Overdependence on countries abroad for medicines

In most African countries, pharmaceutical imports comprise as much as 70–90% of drugs consumed [28]. This overwhelming dependence on importation predisposes vulnerable citizens to shortages. This stems from two major reasons: firstly, there is an immense paucity of in‐country drug‐producing industries and secondly, there is a wide distrust in locally made drugs. The consequence, therefore, is an enormous demand for overseas drugs which cannot match the importable quantities. The resulting shortages from lack of access to specific brands lead to deterioration of illness and an increase in mortality, signalling a poor health index. Moreover, excessive dependence on imported drugs also enables a spike in the production of counterfeit drugs which are often marketed as the original to unsuspecting consumers [32].

This jarring problem also signals ominous consequences on the economy of the Africa region. Overdependence on imported drugs pushes the economy to the edge of trade deficits [27]. It increases the countries’ reliance on foreign political and economic power and consequently triggers inflation, reduced domestic currency values, and interest rates− all of which are indicators of the economic contraction and reduced standard of living.

### Poor supply chains systems

The entire process of drug manufacturing in a different country, the exportation of the final formulation, packaging, and repackaging of the drugs, and eventual sale of the packaged products can be complex. An efficient distribution system is, therefore, needed to ensure drug quality and ease of access. Despite the increased investment in the procurement of drugs, their availability in Africa remains extremely low. Inefficient drug distribution can drive up costs and cause drug stock‐out [33]. The lack of a functioning drug supply chain has been observed to be one of the major causes of poor access to essential medicines. Evidence shows that the supply chain system in Africa is largely weak and unsustainable, laden with lapses and a high incidence of counterfeit drugs, amidst poor regulation [34].

The causes of poor performance of medicines logistics and supply chain systems were identified to include: uncoordinated and unstandardized logistics systems, poor supply chain practices, poor forecasting and data management, stifling of local drug manufacture, regulatory concerns, weak policy implementation, and lack of support for in‐country medical logisticians [33, 35, 36].

### Lack of government investment in pharmaceutical sector

The prevailing government apathy and lack of political will encapsulated by a consistent lack of investment in healthcare affect people's access to drugs [37]. There is a massive shortage of incentives to promote local manufacture of medicines as is evidenced by inadequate funding of research and development, lack of provision of financial support to manufacturers, heavy taxation levied against pharmaceutical industries, and overall inadequate healthcare budgetary allocations [30, 37].

Additionally, there is an inadequate implementation of existing policies such as the Primary Health Care policy in many African regions [38]. This policy aimed at ensuring equity in access to quality healthcare services has suffered severe setbacks due to poor implementation. The primary healthcare systems are rife with fragmented services, a weak referral system, and a huge gap in access to basic healthcare. A vast number of health facilities are located in urban areas, leaving the residents of rural areas with limited healthcare access and resulting in the inadequate spatial distribution of health services and products [39].

Government healthcare financing in Africa is critically low [40]. There is abysmally poor coverage of insurance schemes with a significant number of the population unable to access the services. Thus, there is a disturbingly high un‐pooled source of payment for medical services and products including out‐of‐pocket spending by the populace which accounts for as high as 70% of total health expenditure in the region [37]. Amidst enduring poverty, a worrisome percentage of people are unable to access medicines and healthcare services− a nightmare obstacle towards achieving Universal Health Coverage.

### Unfavourable Manufacturing Conditions

Drug production in Africa is typically done using small plants with low capacity and output [30, 41]. In the context of solid dosage forms, for instance, this limits the capacity to produce enough tablets to achieve economies of scale and lower overall production costs. According to a report by McKinsey, African manufacturers need to produce at least half a billion tablets per year to be cost‐competitive with India [28]. However, the prevailing low manufacturing capabilities often lead to high drug prices and discourages local drug production. Moreover, the utilization and efficiency of manufacturing plants are further exacerbated by high electricity costs, frequent power interruptions, and other infrastructural issues such as poor transportation and logistics systems [30]. Unsurprisingly, these unfriendly manufacturing conditions are likely to outweigh any intended production‐cost advantages of local drug manufacturing. As a result, the affordability of these medicines is adversely affected, as the overhead costs are ultimately transferred to patients and end‐users, obstructing access to affordable medicines. Additionally, compared to European and Asian counterparts, African manufacturing facilities are generally small and lack the required production equipment, with operations that fail to meet international standards [28].

### Limited health workforce

Globally, the crisis of insufficient healthcare workers is most pronounced in Africa, particularly sub‐Saharan Africa [42], thus limiting access to medicines. The region accounts for 36 out of the 57 countries with a critical shortage of health workers, having 2.3 healthcare personnel per 1000 population, a far cry compared to 18.9 and 24.8 in Europe and the Americas, respectively [43]. Expectedly, this has remarkable consequences on healthcare delivery, including access to medicines. The inadequate number of trained health professionals makes it increasingly difficult for medical interventions, such as medicines and vaccines, to be readily available, accessible, and effectively administered by trained and competent personnel [42].

Likewise, the unprecedented exodus of doctors, pharmacists, nurses, and other healthcare workers to developed countries also worsens the already frail health systems of African nations, further impairing access to medicines [44]. Furthermore, there is evidence to show that this worrisome trend may further deteriorate if not addressed promptly and properly. A meta‐analysis revealed that the intention to leave among African nurses was approximately 52% [45], considerably higher compared to Israel (9%) [46], Brazil (22.1%) [47], and Europe (33%) [48]. Given that nurses play a pivotal role in the healthcare system and are primarily responsible for administering medications, this signals a huge impediment in access to medicines and other healthcare services if left unchecked.

### Lack of sustainable health financing mechanism

Many African countries lack proper health financing frameworks needed to achieve UHC, making access to affordable medicines challenging. Despite the 2001 Abuja Declaration in which African leaders agreed to allocate at least 15% of the general government expenditure to health, only five countries (Botswana, Togo, Madagascar, Rwanda, and Zambia) have kept to the commitment [49]. Moreover, twenty‐two (22) African countries do not meet the minimum health expenditure of $44 per capita as defined by the High‐Level Task Force on Innovative International Financing for Health Systems (HLTF), and only three countries (Botswana, Rwanda, Zambia) have met both the Abuja Declaration and the HLFT requirements [49]. As a result, accessing medicines in Africa presents a significant challenge and often leads to catastrophic health expenditure characterized by high financial risks and huge out‐of‐pocket payments which account for over 40% of the total health expenditure in at least 20 countries across Africa [50].

Considering health insurance cover and uptake is low across the continent, many people who need medicines are unable to receive treatment due to the inability to pay. In Nigeria, for instance, only 5% of the population is covered by the National Health Insurance Scheme (NHIS) funded by the government [51]. Given that the NHIS primarily covers persons employed by the federal government, those in the private sector, the self‐employed, and the unemployed which constitute the vast majority of the population are uninsured and lack financial protection. Consequently, in trying to access medicines, many families experience financial hardship, and are pushed below the poverty line [52]. Poor health financing equally represents a significant barrier in optimizing health and treatment outcomes as the accompanying cost of getting medications makes it practically impossible for low‐income patients to have access to the care they need.

### Lack of Infrastructure and technical know‐how

The supply of medicines is mired by inadequate infrastructure in many countries in Africa and has in turn affected access to medicines. A prime example is vaccine supply in Africa. A study shows that at least three in four African countries do not have adequate systems in place to handle vaccine supply [53]. This results in frequent stock‐outs, products getting expired and some, damaged during storage and transit. Additionally, it has been shown that the most essential factors influencing efficient inventory management and transport and distribution of medicines are human resource capacity and process management [35]. However, many are not able to access medicines in Africa mainly due to: low number of skilled personnel, logistics being handled by untrained personnel, poor process management, poor transparency and communication levels, poor assessment of performance level [35]. There is also a dearth of skilled local talents that have knowledge and expertise in drug research and development, modern manufacturing techniques, and the use of sophisticated industrial tools and machines needed to boost drug manufacturing [41].

### Lack of effective pricing and price regulations

In the year 2007, the 55 members of the African Union endorsed the Pharmaceutical Manufacturing Plan for Africa, a non‐binding framework ‘to pursue, with the support of our partners, the local production of generic medicines on the continent’. Despite this, the high prices of essential medicines continue to be a major challenge limiting access to care to a large proportion of poor populations in Africa [54]. In countries such as Zambia, Nigeria, Senegal and Tunisia, frequently use generic drugs like paracetamol can cost up to 30 times more than its price in the UK and USA [55]. Many African countries are also not able to control and regulate the prices of medicines, and they are procuring essentials medicines at high prices than the internationally recognized price [55, 56, 57]. For instance, imported medicines approved by the drug regulatory agency in Sudan were ten times more costly than the internationally standard reference price [56]. The lack of effective pricing policies and regulations has made prices in the private sector pharmacies to remain higher than international prices, thus making treatments for chronic diseases unaffordable to many in developing countries [55].

The WHO has proposed several cost‐containment measures to prevent the further rise of treatment costs including the regulation of mark‐ups in the pharmaceutical supply chain, use of reference pricing, exemptions or reduction of tax for essential medicines, promoting the use of generics, promoting price transparency, and also the use of health technology assessment to notify pricing and reimbursement decisions [58]. However, the lack of supporting frameworks, technical capacity, relevant data have hindered the effective implementation of these cost‐containment strategies in most developing countries including Africa [59]. Therefore, a regulatory mechanism should be established in African countries, where such systems are not in existence to ensure prices of essential medicines are thoroughly monitored and controlled. Additionally, the use of cheap generic medicines should continue to be encouraged.

### Circulation of fake and counterfeit medicines

Another crucial challenge to be addressed is the increased circulation of fake and counterfeit medicines. Counterfeit or fake medicines are medicines which are deliberately and fraudulently mislabelled with respect to identity and/or source [60]. A WHO report stated that Africa alone accounts for 42% of globally detected cases of counterfeit and substandard medical products [32]. Coupled with poor regulation policies and widespread corruption, fake drugs abound in markets, the harm from which people suffer and die in dreadful numbers. The borders of the region are very porous leading to smuggling of fake, substandard, counterfeit and falsified medicines. The increase in circulation of fake and counterfeit medicines in Africa has posed a great challenge in accessing quality medicines and this has caused waste of funds by consumers, and loss of revenue to pharmaceutical companies up to 40% [61]. To circumvent this challenge, more deliberate effort and attention need to be put in curtailing the silent and devastating effects of fake and counterfeit medicines circulating in Africa.

### Patent issues and limited investment on research and development

Restrictions in access to patents and inability of local manufacturers, researchers and scientists to gain patents is a barrier to accessing medicines in Africa. Pharmaceutical industries in many African countries are not optimally manufacturing new medicines due to low investment in research and development. Disease burden is high in most African countries because of inadequate funding for innovation [62]. Evidence has also shown that African governments struggle to fulfil their commitments to research and development, such that only a few kinds of research are being carried out and the ones carried out are not put into practice [30]. There is also low intra‐African research collaboration due to low funding and incentives within the continent and this encourages under‐research of some diseases prevalent in Africa and ultimately affecting the production of new medicines [63].

## RECOMMENDATIONS

We discussed recommendations to improve access to medicines. See Table 1 for current efforts and challenges‐specific recommendations.

**TABLE 1:** Overview of the current efforts by African governments and other stakeholders and challenges‐specific recommendations to strengthen access to medicines in Africa

Challenges facing access to medicines in Africa	Current efforts	Specific recommendations
High burden of infectious and non‐infectious diseases	Agencies involved in strengthening healthcare system to effectively respond to diseases and public health emergencies.	Improve disease surveillance, health promotion and inter‐sectoral collaboration Poverty alleviation programmes
Limited pharmaceutical industries and high costs of raw materials	National Medicines Regulatory Medicines Authorities (NMRAs) focus on regulation of medical products, rather than expanding and strengthening pharmaceutical industries	African Medicines Agency (AMA) to establish additional pharmaceutical companies Government should subsidize raw materials including APIs. Research on cost‐effective and readily available raw materials Government grants to local drug manufacturers.
Overdependence on countries abroad for medicines	Despite recommendations from researchers, and other stakeholders, government response and political will in this regard is still limited.	Invest more in ensuring increase in the presence of more pharmaceutical companies. Private‐public partnerships in this regards would be beneficial. The presence of the proposed AMA should also be enhanced across the continent.
Poor supply chains systems	Many African countries have its NMRAs with the role of ensuring effective and efficient drug supply chain systems. However, only 15% of the NMRAs have a legal mandate to perform all critical regulatory functions [80].	Revitalization of all the NMRAs. Invest in capacity building of medicines regulatory officers. Developing structural models to address any local barriers, for example, corrupt practices. Strengthening digitalization of supply chain activities
Lack of government investment in pharmaceutical sector	Stakeholders continue to advocate for more investment from the government to revitalize the pharmaceutical sector on the continent [15, 23].	Recognition of the pharmaceutical sectors with direct investment by the government. Increase in political will
Unfavourable manufacturing conditions	High electricity costs, frequent power interruptions, and other infrastructural issues such as poor transportation continue to persist despite all the policy recommendations.	Invest in making the continent conducive for pharmaceutical manufacturing, towards attracting more international investors.
Limited health workforce	Community health workers (CHWs) and patent medicines vendors have played active role in improving access to medicines [81, 82]. Some countries have also invested in improving access to medical, pharmacy and health education across tertiary institutions.	Engage CHWs and patent medicines vendor as well as upskilling them. Specialized practical training in pharmaceutical manufacturing, quality assessment, process control, and the use of modern equipment should be incorporated into the core curriculum of pharmacy schools. Improve access to education through educational grants
Lack of sustainable health financing mechanism	Effort is seen in advancing health insurance systems on the continent. However, there is still a huge concern regarding coverage. For example, the insurance coverage in Kenya is 19%, and Nigeria is 5%.	Strengthen health insurance systems. Increase in budgetary allocation to health in African countries Revitalization of primary health care systems remain pertinent.
Lack of infrastructure and technical know‐how	Even though more still needs to be done, pharmaceutical companies, with limited support from governments, are involved in strengthening manufacturing capacity through training, and international collaborations.	Invest directly in supporting manufacturing industries through capacity building, improved access to infrastructure, and building local talents.
Lack of effective pricing and price regulations	Even though it is recommended that African countries should have agency aimed at regulating medicines prices, most do not perform the role.	Set up an initiative that is aimed at regulating drug prices with evidence‐based pillars in making such decisions.
Circulation of fake and counterfeit medicines	The medicines regulatory landscape is undergoing transformation with Nigeria, Egypt, Tanzania and Ghana having NMRAs operating at WHO maturity level 3. The NMRA's surveillance effort to reduce the circulation of fake and counterfeit medicines are, however, undermined.	Invest in strengthening medicines supply chain systems. Active and passive surveillance of drugs in circulation should be enhanced. Employing people‐centred technological innovation. Close open drug market.
Patent issues and limited investment on research and development	There is a growing discussions and actions on the need to invest in herbal medicine research. Struggle to protect patents persists and advocacy action is still growing in this regard.	Massive investment in pharmaceutical research. Initiative should be enhanced to protect African‐based patents.

Abbreviations: AMA, African Medicines Agency; APIs, Active Pharmaceutical Ingredients; CHWs, Community Health Workers; NMRAs, National Medicines Regulatory Medicines Authorities; WHO, World Health Organization.

### Direct investment on the health systems

A key component of good health systems is uninterrupted access to effective, safe, quality, and affordable medicines [64]. While efforts and investments are being made to curb the double burden of diseases on the continent, resources and financial investment towards ensuring access to medicines need to be prioritized. Currently, most African countries have been falling short of the 2001 Abuja Declaration since its inception, where at least a minimum of 15% of the total budget should be allocated to health; however, out‐of‐pocket payment remains the major means of financing health services including medicine purchases [37]. This is a wake‐up call for governments and national health authorities in Africa to ensure investment in the overall health systems as well as strengthen health insurance systems on the continent.

### Increased investment in herbal medicine research

Herbal medicines have stood the test of time since time immemorial. They are generally more accessible, more affordable, and more acceptable to the local population. About 80% of the population in Africa use herbal medicines in one form or the other and about 25% of drugs used worldwide are derived from herbs [65]. Although largely non‐prescriptive, the sources of information are always relatives, friends, or herbal specialists. The materials used are always easily accessible and inexpensive. The need for standardization and complete commercial exploitation of the potential benefits from herbal and traditional medicines thus becomes imperative [66]. Africa has demonstrated a growing interest in promoting and preserving the traditional knowledge on the use of herbal medicines. This is active in Senegal and Dakar through the activities of non‐Governmental organizations such as the Association for the Promotion of Traditional Medicines (PROMETRA) [67]. The Centre for Plant Medicine Research (CPMR) was established in 2010 in Ghana to promote, encourage and regulate scientific research knowledge and development in the field of plant medicine [68]. The initiative of preserving the local culture and sharing of their knowledge to the global health as a way of increasing the countries market share has taken root in Nigeria [69].

Despite this huge potential, there is low investment in traditional medicine across Africa, the safety and efficacy of herbal products are unestablished, and scientific researches to discover new herbal products and technologies to harness them are hushed in low tones. Therefore, it is expedient that governments and collaborating partners increase finances on herbal medicine; policies that integrate herbal medicine into national health systems must be implemented and safeguarded; significant research must be encouraged and devoted to herbal medicine including rapid genetic sequencing that offers vast resource for discovery of newer agents.

### Strengthening access to medicines through public‐private partnerships

Public‐Private Partnership is a key element to be considered in improving access to medicines in Africa. For instance, a community‐based distribution model of enhancing access to medicines [70] will require public‐private partnership. The collaboration between governments institutions, private sectors, public health institutions and pharmaceutical companies can help to strengthen research capacity and improve local manufacture [71]. This is worth exploring on the continent to improve access to medicines.

### Establishing effective pharmaceutical drug policies

Since most of the medicines used in Africa are imported, drug policies that target the improvement of local production of drugs would greatly improve the access to medicines in the region. Historically, poor implementation of existing policies has led to a shortage of essential medicines and will continue to do so unless deliberate interventions are made. Such interventions should include strengthening the drug policies to support local expertise in the manufacture and quality control of drugs. Adequate training and funding of drug regulatory agencies in the region should be put in place as they are critical to eliminating the distribution of fake and counterfeited medicines. Collaboration between countries in the region to develop a system to limit the free movement of fake and adulterated drugs would help to support local manufacturers. A robust system to enforce the policies is equally important to ensure strict compliance. Smooth development and enforcement of drug policies have the potential to bring the continent one step closer to achieving access to safe and quality medicines. In addition, harmonization of African Medicines Agencies is also a very important step to address the anaemic access to medicines on the continent [72].

### Establishing more in‐continent pharmaceutical companies

There is currently an increasing interest among African and international policy makers, researchers, and some donors and suppliers, to improve local African pharmaceutical supply by supporting new and existing pharmaceutical companies. This is much‐needed to improve the continent's capacity to produce medicines for its populace. Investing in establishing more in‐continent pharmaceutical companies will improve access to medicines which is crucial to attain universal health coverage in Africa [73].

### Investment in industrial pharmacy education and capacity building on the continent

A commitment to investment in industrial pharmacy education can help to bridge gaps in access to medicines in Africa through development of human resources and advocacy [74, 75]. Specialized practical training in pharmaceutical manufacturing, quality assessment, process control, and the use of modern equipment should be incorporated into the core curriculum of pharmacy schools to stimulate interest in industrial pharmacy, improve local manufacturing capacity and galvanize renewed actions towards making medicines more available, accessible, and affordable to Africans. Additionally, African‐based pharma and biotech companies should make strides to improve existing systems and structures, particularly in research and development, in order to meet the ever‐growing healthcare needs of the teeming African population.

### Evidence‐based adaptation of WHO essential medicines lists for local use

The World Health Organization publishes Model List of Essential Medicines, which is updated every 2 years since 1977 when it was first published [76]. The list contains the medications which are considered to be most effective and safe to meet the most important needs in a health system. Even though many African countries frequently use the list to help develop their own local lists of essential medicines, often times the locally adapted essential lists are not evidence‐based [77]. We recommend that African countries should engage in active research to understand the local medicines need of their population and adapt or modify the essential medicines lists based on this.

### Rapid ratification of the proposed African medicines agency treaty

The African Medicines Agency's (AMA), which is a component of the African Union Model Law, has a vision to ensure that all Africans have access to quality‐assured, efficacious, safe and affordable medical products, that meet globally recognised standards, for major diseases or prioritized conditions [72, 78]. Ultimately, instead of having 55 National Medicines Regulatory Agencies (NMRAs) on the African continent, each with its own regulatory requirements, the AMA intends, among other goals, to result in streamlined regulatory processes in order to enable the timely evaluation and subsequent registration of medical products [79]. The African Medicines Regulatory Harmonization initiative is the foundation for the establishment of the African Medicines Agency [72]. To date, 17 member states of the African Union (Algeria, Benin, Burkina Faso, Cameroon, Chad, Gabon, Ghana, Guinea, Mali, Mauritius, Namibia, Niger, Rwanda, Seychelles, Sierra Leone, Tunisia and Zimbabwe) have ratified the Treaty for the Establishment of the African Medicines Agency and deposited the legal instrument of ratification to the Commission [78]. In total, 26 member states (Algeria, Benin, Burundi, Cameroon, Chad, Cote d'Ivoire, Egypt, Gabon, Ghana, Guinea, Madagascar, Mali, Mauritius, Morocco, Niger, Rwanda, Republic of Congo, Saharawi Arab Democratic Republic, Senegal, Seychelles, Sierra Leone, Tanzania, Togo, Tunisia, Uganda and Zimbabwe) have signed the treaty [78]. We urge African countries to sign and ratify the Treaty for the Establishment of the African Medicines Agency in the interest of public health, safety and security. In addition to this, sustainable financing mechanism and political commitment across African countries to ensure the success of AMA is pertinent.

## CONCLUSION

Without reliable access to medicine systems, the healthcare system is incomplete and good health and well‐being cannot be guaranteed. It is clear that access to medicines in Africa faces numerous challenges and it is pertinent for the continent to address these issues as it is geared towards strengthening its health systems for universal health coverage. We call on African governments and national health authorities to prioritize efforts and initiatives to strengthen access to medicines using tailor‐made mechanisms and country‐compatible strategies.

## CONFLICT OF INTEREST

The authors declare no competing interests.

## ETHICS STATEMENT

There is no need for ethical approval.

## AUTHOR CONTRIBUTIONS

Yusuff Adebayo Adebisi conceptualized the idea of the paper. A data gathering and a drafting group was composed of Yusuff Adebayo Adebisi, Ifechukwu Benedict Nwogu, Aishat Jumoke Alaran, Abubakar Olaitan Badmos, Adeboye Olakunle Bamgboye, Basirat Oluwadamilola Rufai, Osaretin Christabel Okonji, Malik O. Malik, John Oluwafemi Teibo, and Samah Faisal Abdalla. All authors contributed in the analysis of the data and the revision iterations. Final rewriting and revision were done by Yusuff Adebayo Adebisi, Don Eliseo Lucero‐Prisno III, Mohamed Samai, and Wuraola Akande‐Sholabi. All co‐authors agreed to the final draft of the paper.

## Data Availability

This is a review article and all sources used were duly cited.
